# Autophagy mediates cell cycle response by regulating nucleocytoplasmic transport of PAX6 in limbal stem cells under ultraviolet-A stress

**DOI:** 10.1371/journal.pone.0180868

**Published:** 2017-07-10

**Authors:** Maria Laggner, Andreas Pollreisz, Gerald Schmidinger, Ursula Schmidt-Erfurth, Ying-Ting Chen

**Affiliations:** Department of Ophthalmology & Optometry, Medical University of Vienna, Vienna, Austria; Univerzitet u Beogradu, SERBIA

## Abstract

Limbal stem cells (LSC) account for homeostasis and regeneration of corneal epithelium. Solar ultraviolet A (UVA) is the major source causing oxidative damage in the ocular surface. Autophagy, a lysosomal degradation mechanism, is essential for physiologic function and stress defense of stem cells. PAX6, a master transcription factor governing corneal homeostasis by regulating cell cycle and cell fate of LSC, responds to oxidative stress by nucleocytoplasmic shuttling. Impaired autophagy and deregulated PAX6 have been reported in oxidative stress-related ocular surface disorders. We hypothesize a functional role for autophagy and PAX6 in LSC’s stress response to UVA. Therefore, human LSC colonies were irradiated with a sub-lethal dose of UVA and autophagic activity and intracellular reactive oxygen species (ROS) were measured by CYTO-ID assay and CM-H_2_DCFDA live staining, respectively. Following UVA irradiation, the percentage of autophagic cells significantly increased in LSC colonies while intracellular ROS levels remained unaffected. siRNA-mediated knockdown (KD) of *ATG7* abolished UVA-induced autophagy and led to an excessive accumulation of ROS. Upon UVA exposure, LSCs displayed nuclear-to-cytoplasmic translocation of PAX6, while ATG7KD or antioxidant pretreatment largely attenuated the intracellular trafficking event. Immunofluorescence showing downregulation of proliferative marker PCNA and induction of cell cycle regulator p21 indicates cell cycle arrest in UVA-irradiated LSC. Abolishing autophagy, adenoviral-assisted restoration of nuclear PAX6 or antioxidant pretreatment abrogated the UVA-induced cell cycle arrest. Adenoviral expression of an ectopic PAX gene, PAX7, did not affect UVA cell cycle response. Furthermore, knocking down PAX6 attenuated the cell cycle progression of irradiated ATG7KD LSC by de-repressing p21 expression. Collectively, our data suggest a crosstalk between autophagy and PAX6 in regulating cell cycle response of ocular progenitors under UVA stress. Autophagy deficiency leads to impaired intracellular trafficking of PAX6, perturbed redox balance and uncurbed cell cycle progression in UVA-stressed LSCs. The coupling of autophagic machinery and PAX6 in cell cycle regulation represents an attractive therapeutic target for hyperproliferative ocular surface disorders associated with solar radiation.

## Introduction

The corneal epithelium, an indispensable prerequisite for visual acuity, is postnatally maintained and regenerated by a pool of adult stem cells, termed limbal stem cells (LSC) [1–4]. Solar ultraviolet A (UVA) is a major environmental hazard causing acute photodamage in cornea and chronic exposure is often associated with hyperproliferative, yet degenerative ocular surface diseases, such as pterygium [5–7]. Cells respond to UVA stress by activation of antioxidant signaling pathways, dynamic regulation of cell cycle or apoptosis. To date, key cellular and molecular signaling events driving LSC’s stress response remain unclear in UVA-related ocular pathology. Autophagy, a lysosomal degradation system, is essential for maintenance of stem cell characteristics, including self-renewal, differentiation and quiescence [8–11]. Accumulating evidence suggests that autophagy contributes to cellular defense mechanisms in somatic stem cells under various types of stress [12,13]. Whether autophagy plays a role in LSC’s stress response to UVA remains elusive.

Paired-box protein 6 (PAX6) is a master transcription factor guiding corneal morphogenesis and homeostasis by regulating cell cycle and fate of tissue progenitor cells [14,15]. Recent reports suggest that PAX6 is implicated in corneal wound healing [16] and inflammatory response [17,18], both of which are cellular events with a transient surge of reactive oxygen species (ROS). Interestingly, Ou *et al*. demonstrated that PAX6 in corneal epithelial cells responded to oxidative stress by nuclear-to-cytoplasmic translocation [19]. However, the molecular mechanism and the physiological/pathological significance of the oxidative stress-triggered PAX6 relocation remains undetermined. In this study, we sought to characterize LSC’s cellular response to UVA radiation and explored the potential role(s) of autophagy and PAX6 in the identified stress response.

## Materials and methods

### Cell culture of primary human LSC

This research has been approved by the Ethical Committee of the Medical University of Vienna (IRB approval number MUW 1578/2013). Human corneal tissues procured in MUW cornea bank not suitable for corneal transplantation were used in the current study. Limbal epithelial sheets from corneoscleral rims were surgically isolated after overnight incubation in dispase II (10.7 U/mL, from *Bacillus polymyxa*, Sigma-Aldrich, St. Louis, MO) at 4°C and subjected to trypsin digestion for 10 mins at 37°C. Cells were seeded at densities ranging from 100 to 400 cells/cm^2^ and cultured up to 12 days. Colonies were expanded in serum- and feeder cell-free conditions in Keratinocyte-Serum Free Medium (KSFM, Gibco, Thermo Fisher Scientific Inc., Waltham, MA) supplemented with 5 ng/mL human recombinant epidermal growth factor and 50 μg/mL bovine pituitary extract (both Gibco).

### siRNA Transfection

siRNA oligonucleotide duplexes targeting human *ATG7* and *PAX6* were obtained from Ambion (Thermo Fisher Scientific Inc.). Sense and antisense sequences were as follows: *ATG7* Knockdown (KD) #1 sense 5’-CGCUUAACAUUGGAGUUCAtt-3’ antisense 5’-UGAACUCCAAUGUUAAGCGag-3’, KD#2 sense 5’-GGAACACUGUAUAACACCAtt-3’ antisense 5’-UGGUGUUAUACAGUGUUCCaa-3’; *PAX6*, KD#1 sense 5’-GGCAAUCGGUGGUAGUAAAtt-3’ antisense 5’-UUUACUACCACCGAUUGCCct-3’, KD#2 sense 5’-CCAACUCCAUCAGUUCCAAtt-3’ antisense 5’-UUGGAACUGAUGGAGUUGGta-3’.Controls were transfected with two scrambled siRNAs (SCR) with no complementarity to any known human gene product (Silencer Select Negative Controls, Ambion). For a 60-mm petri dish, 12.5 pmol of each siRNA and 6.25 μl lipofectamine (RNAiMAX Reagent, Thermo Fisher Scientific Inc.) were used to transfect colonies on clonal day 8–10 at 70% confluence. Transfection mixture was removed after overnight incubation and cells were cultured for additional three days in KSFM prior to UVA irradiation and further experiments. Transfection efficiency was assessed by quantitative PCR, western blot and immunofluorescene analysis.

### *PAX6* and *PAX7* overexpression

Pre-packaged human adenovirus (dE1/E3 serotype 5, Vector Biolabs, Malvern, PA) expressing the human *PAX6* gene under control of the cytomegalovirus (CMV) promoter (Ad-CMV-PAX6) was used to ectopically express PAX6 in LSC colonies. To study whether the observed PAX6 gene functions were ocular tissue-specific, experiments of ectopic PAX7 gene expression in LSC colonies were performed in parallel. Adenoviral cassettes carrying viral backbones and an empty CMV promoter (Ad-CMV-null) served as controls. Colonies were infected on clonal day 10 with an MOI of 50 overnight. Cells were cultured for additional two days in adenovirus-free KSFM for protein expression before experiments were performed.

### UVA irradiation

Sellamed 3000 UVA-1 irradiation device (Sellas, Ennepetal, Germany) was used to generate radiation filtered for the emission of UVA light in the spectral range of 340–440 nm. During irradiation, cells were transferred to Dulbecco’s Phosphate Buffered Saline (DPBS, Gibco). A non-lethal dose of UVA titrated to the irradiance of 20 J/cm^2^ was used throughout all experiments to study PAX6’s regulation on cell cycle progression in UVA-stressed LSCs (S1 Fig).

### Anti-oxidant treatment and ROS detection

Cells were treated with an antioxidant mixture (10 μM α-tocopherol and 15 mM N-acetyl-L-cysteine, both Sigma-Aldrich) for 16 hours prior to UVA irradiation. Cells treated with 20 μM H_2_O_2_ for 6 hours served as positive controls for oxidative stress. ROS levels were visualized using CM-H_2_DCFDA (10 μM; Molecular Probes, Eugene, OR) according to the manufacturer’s instructions. Live cell imaging was performed by laser scanning confocal microscopy 30 minutes after exposure to UVA. For quantification, mean fluorescence intensity of LSC colonies was determined.

### Autophagosome and autophagic flux measurement

Autophagy activity was determined by autophagosome formation and autophagic flux. Autophagosomes were visualized 6 hours after UVA exposure using a cationic amphiphilic tracer dye to stain autophagosomal vesicles (CYTO-ID, Enzo Life Sciences, Farmingdale, NY). Cells treated with 10 μM rapamycin (from *Streptomyces hygroscopicus*, Sigma-Aldrich) for 6 hours served as positive controls. Autophagic cells were defined as cells displaying more than five perinuclear puncta [20,21]. A minimum of 250 clonal cells were counted in each sample and data are presented as percentage of autophagic cells per colony. Basal and UVA-stimulated autophagic flux were determined by western blot analysis of lipidated form of LC3B (LC3B-II). LC3B-II expression in the absence or presence of autophagic flux inhibitor Bafilomycin A1 (BafA1, 100 nM from *Streptomyces griseus*, Sigma-Aldrich) with or without UVA irradiation was assessed at different time points up to 24 hours [22]. Data were normalized to glyceraldehyde-3-phosphate dehydrogenase (GAPDH) expression levels for densitometric analysis.

### Apoptosis detection

Apoptotic cells were quantified 6 hours after UVA irradiation using terminal deoxynucleotidyl transferase-mediated dUTP nick end labeling assay (TUNEL; Click-iT TUNEL Imaging Assay, Thermo Fisher Scientific Inc.) according to the manufacturer’s protocol.

### Reverse transcription-quantitative polymerase chain reaction (RT-qPCR)

Total RNA was isolated using RNeasy Plus Mini Kit (Qiagen, Hilden, Germany) and reverse transcribed using iScript cDNA Synthesis Kit (Bio-Rad Laboratories, Inc., Hercules, CA). qPCR was performed using TaqMan Gene Expression Assays and Gene Expression Master Mix (both Applied Biosystems, Foster City, CA). Amplification reactions were carried out according to a standardized qPCR program of the ABI Prism 7500 Fast Real-Time PCR System (Perkin Elmer, Applied Biosystems). Data were analyzed using the “delta-delta method” as suggested by Applied Biosystems and Pfaffl [23]. Results display averaged relative quantification (RQ) target gene expression normalized to *GAPDH* values of at least three biological repeats.

### Immunofluorescence (IF) confocal microscopy

For immunofluorescent staining, cells were fixed in 4% (v/v) formaldehyde and subsequently immersed in DPBS containing 0.1% (v/v) Triton X-100 for permeabilization. Samples were blocked using serum-free Protein Block (Dako, Glostrup, Denmark). Antibody diluent (Dako) was used to dilute primary and secondary antibodies. Mouse and rabbit IgG served as isotype controls (Vector Laboratories, Burlingame, CA). 4,6-diamidino-2-phenylindole dilactate (DAPI) and Hoechst 33342 (both Molecular Probes) were used as nuclear counterstains for fixed and viable cells, respectively. Images were acquired using an inverted laser scanning confocal microscope (LSM780, Carl Zeiss GmbH, Oberkochen, Germany) equipped with a 405-nm laser diode and an argon ion laser capable of providing an illumination of 488 nm. A helium-neon laser was used to generate an excitation wavelength of 594 nm. Fluorescent signals were detected by a 32-channel spectral detection unit (gallium-arsenide-phosphide detector 416–728 in 8.3 nm steps, Carl Zeiss). Imaging was routinely performed with a 20x air objective (numeric aperture 0.8) and a 63x oil lens (numeric aperture 1.4) was used to detect autophagosomal vesicles using ZEN software (2009, Carl Zeiss). Image processing software ImageJ and Fiji (Laboratory for Optical and Computational Instrumentation, Madison, WI) were used for image analysis [24,25].

### Western blotting (WB)

Proteins of LSC colonies were isolated with a cell extraction buffer (Invitrogen, Carlsbad, CA) supplemented with protease inhibitor cocktail (Roche, Basel, Switzerland). Forty μg total protein provided with loading buffer (Bio-Rad Laboratories, Inc.) and 100 mM dithiothreitol were electrophoretically separated on polyacrylamide gels (Excel-Gel SDS 8–18 Gradient, GE Healthcare, Little Chalfont, UK). For LC3B-I/II immunoblotting, a precast 4–20% gradient gel (Criterion TGX, Bio-Rad Laboratories, Inc.) was used. Proteins were transferred to a nitrocellulose membrane (0.2 μm pore size, Invitrogen). Five % (w/v) nonfat dried milk powder and 2% (w/v) bovine serum albumin (BSA, Sigma-Aldrich) in tris buffered saline containing 0.05% (v/v) Tween20 was used for membrane blocking and as antibody diluent. Peroxidase-conjugated secondary antibodies were visualized using Novex ECL (Invitrogen). Chemiluminescence was detected using ChemiDoc XRS system with Image Lab software (version 5.2.1, Bio-Rad Laboratories, Inc.).

### Antibodies

Rabbit polyclonal antibody (Rb pAb) against ATG7 (1:100 for IF, 1:1000 for WB, Abgent, Inc., San Diego, CA), rabbit monoclonal antibody (Rb mAb) against GAPDH (1:1000, Cell Signaling Technology, Danvers, MA), Rb pAb against LC3B (GeneTex, Inc., Irvine, CA), mouse monoclonal antibody (Ms mAb) against p21 (1:50; Santa Cruz Biotechnology, Dallas, TX), Ms mAb against PCNA (1:100, Cell Signaling Technology), Rb pAb against PAX6 (1:300 for IF, 1:1000 for WB; BioLegend, San Diego, CA), AlexaFluor 488-conjugated Donkey Anti-Rb IgG (H+L) (1:1000), AlexaFluor 594-conjugated Donkey Anti-Ms IgG (H+L) (1:1000; both Life Technologies), Peroxidase-conjugated IgG Fraction Monoclonal Ms Anti-Rb IgG, Light Chain Specific (1:5000; Jackson ImmunoResearch Inc., West Grove, PA).

### Statistical analysis

Data represent average of at least 3 independent experiments. For IF data analysis, at least 10 colonies per condition were imaged, unless specified otherwise. Statistical analysis was performed using non-paired Student’s *t* test, one-way ANOVA, Sidak’s, Dunnett’s and Tukey’s multiple comparisons (Graphpad Prism 6.05, GraphPad Software, Inc., La Jolla, CA). Data are shown as means ± s.e.m. with *p* < .05 considered statistically significant.

## Results

### Validation of ATG7KD as an *in vitro* model of autophagy-deficient human LSC

**Fig 1:**
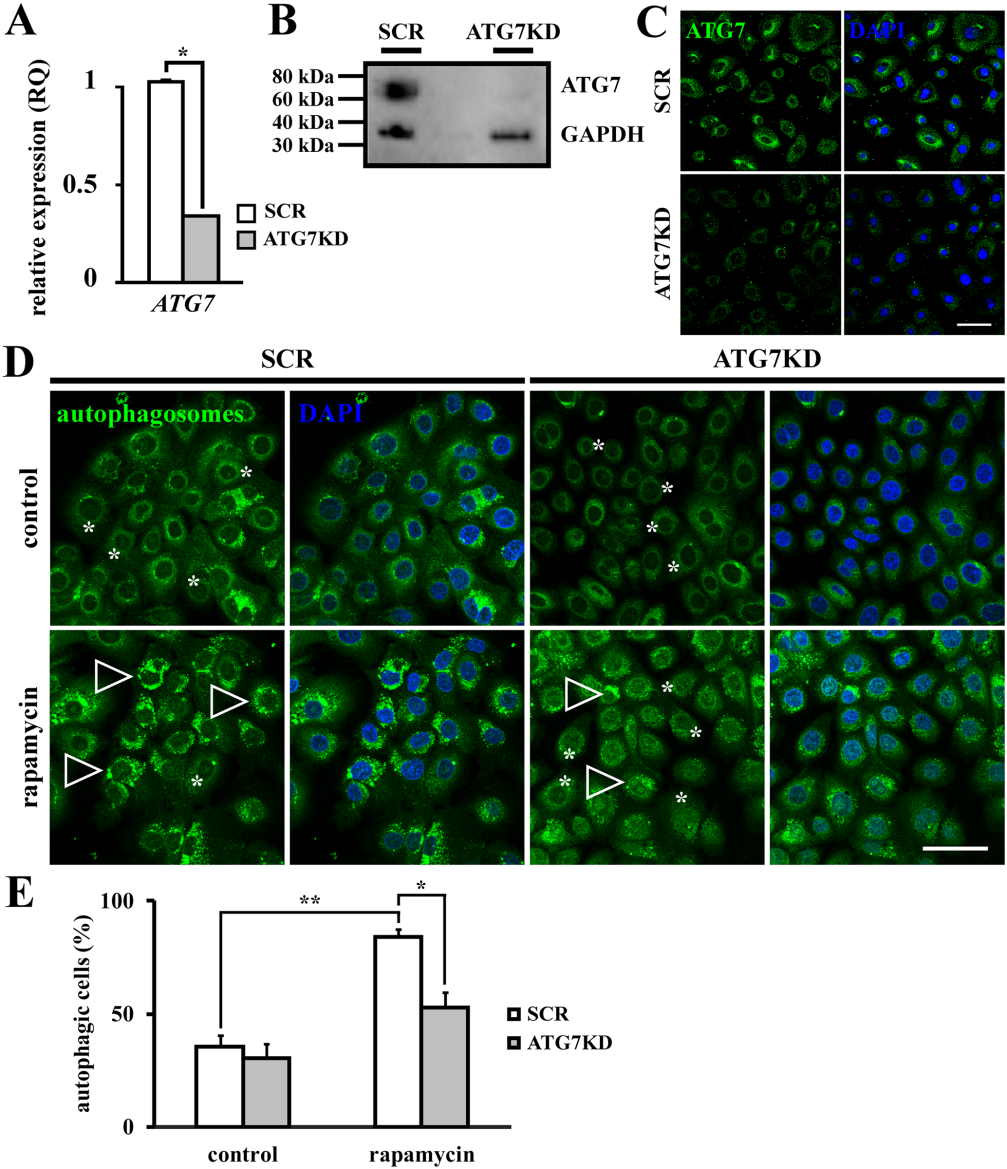
Validation of ATG7KD as an *in vitro* model of autophagy-deficient human LSCs. (A) Knockdown efficiency of *ATG7* verified by qPCR analysis. (B) Western blot analysis of ATG7 expression in SCR and ATG7KD LSC colonies. (C) ATG7 expression in SCR and ATG7KD LSCs assessed by immunofluorescence. Scale bar, 50 μm. (D) Representative micrographs of autophagosome staining by CYTO-ID assay. Arrowheads indicate autophagosomal vesicles, asterisks denote diffuse pattern of autophagic components. Scale bar, 50 μm. (E) Quantification of autophagosomes in SCR and ATG7KD LSCs in response to rapamycin treatment. *p < .05, **p < .01.

In order to generate autophagy-deficient human LSC *in vitro*, colonies during exponential growth phase (clonal days 8–10) were transfected with two siRNAs targeting human homolog of *autophagy-related gene 7 (ATG7)* while controls received scrambled siRNAs not complementary to any human gene sequence (SCR). The efficiency of siRNA-mediated ATG7KD was measured at both mRNA and protein levels. Quantitative PCR demonstrated a 65.1 ± 1.6% reduction of *ATG7* mRNA expression in ATG7KD compared to SCR control (*p* < .05) (Fig 1A). Western blot analysis and immunofluorescence verified KD efficiency of ATG7 in ATG7KD LSC at protein levels compared to SCR transfected controls (Fig 1B & 1C). To validate the functional abolishment of autophagy in ATG7KD LSCs, cellular activity of autophagosome formation in response to rapamycin, an autophagy inducer, was studied. Rapamycin treatment significantly increased the percentage of autophagic cells from basal autophagic levels of 35.7 ± 4.9% to the inductive level of 84.1 ± 4.1% in SCR (*p* < .01), while no inductive effect of rapamycin was observed in ATG7KD LSCs (30.2 ± 6.4% in non-treated ATG7KD versus 52.9 ± 8% in rapamycin-treated ATG7KD, *p*>.05) (Fig 1D & 1E). Taken together, these data validate a successful blockage of inductive autophagy by siRNA-mediated KD of *ATG7* in our LSC clonal culture.

### UVA activates autophagy by increasing autophagic flux

**Fig 2:**
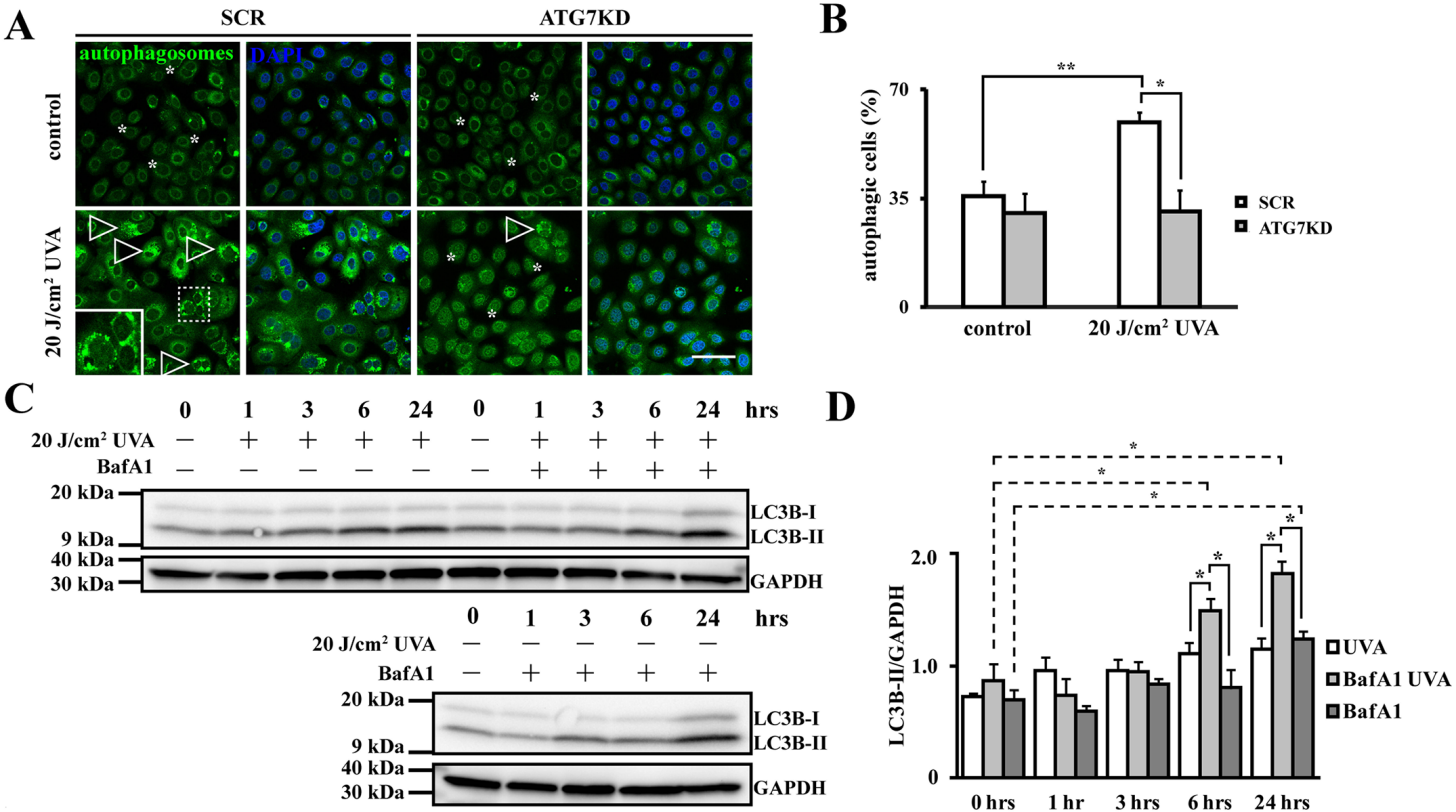
Autophagy is activated during LSC’s stress response to UVA. (A) Representative images of autophagosomes in ATG7KD LSCs under UVA stress. Arrowheads show autophagic cells, asterisks indicate absence of autophagosomes. Scale bar, 50 μm. (B) Quantification of cells with autophagic activity in response to UVA. (C) Representative western blot image of autophagic flux in UVA-irradiated LSC colonies in absence and presence of BafA1, with or without UVA. LC3B-I and II were detected by immunoblotting at indicated time points. (D) Densitometric analysis of LC3B-II expression normalized to GAPDH. n = 3, *p < .05.

To test whether autophagy mediates LSC’s ultraviolet stress response, formation of autophagosomal structures was assessed 6 hours after exposure to 20 J/cm^2^ UVA by CYTO-ID assay. UVA radiation induced autophagy in SCR LSCs evidenced by an increase in autophagic cells from 35.7 ± 4.9% to 59.4 ± 3.3% compared to non-irradiated controls (*p* < .01) (Fig 2A & 2B). In contrast, the number of autophagic cells remained at baseline levels after UVA irradiation in ATG7KD LSC in comparison to untreated counterparts (30.2 ± 6.4% in ATG7KD and 30.8 ± 6.8% after UVA, *p*>.05). Data indicate that UVA increases autophagosomes in an ATG7-dependent mechanism.

To determine whether the observed autophagosome increase in irradiated SCR colonies results from UVA-impaired autophagosomal clearance or UVA-stimulated autophagic flux, we then employed BafA1, an autophagolysosomal inhibitor, to measure autophagic flux at homeostatic state and under UVA stress. In non-irradiated LSCs, LC3B-II was found elevated after 24 hours of BafA1 treatment (*p* < .05 compared to drug vehicle-treated group), indicative of a low homeostatic autophagic flux in LSC colonies (Fig 2C & 2D). In contrast, the elevation of LC3B-II started as early as 6 hours after UVA irradiation and remained elevated at 24 hours in BafA1-treated LSCs (*p* < .05 compared to BafA1-treated, non-irradiated group), suggesting that UVA activates autophagy in LSCs by increasing autophagic flux.

### Autophagy regulates intracellular ROS levels post-UVA irradiation

**Fig 3:**
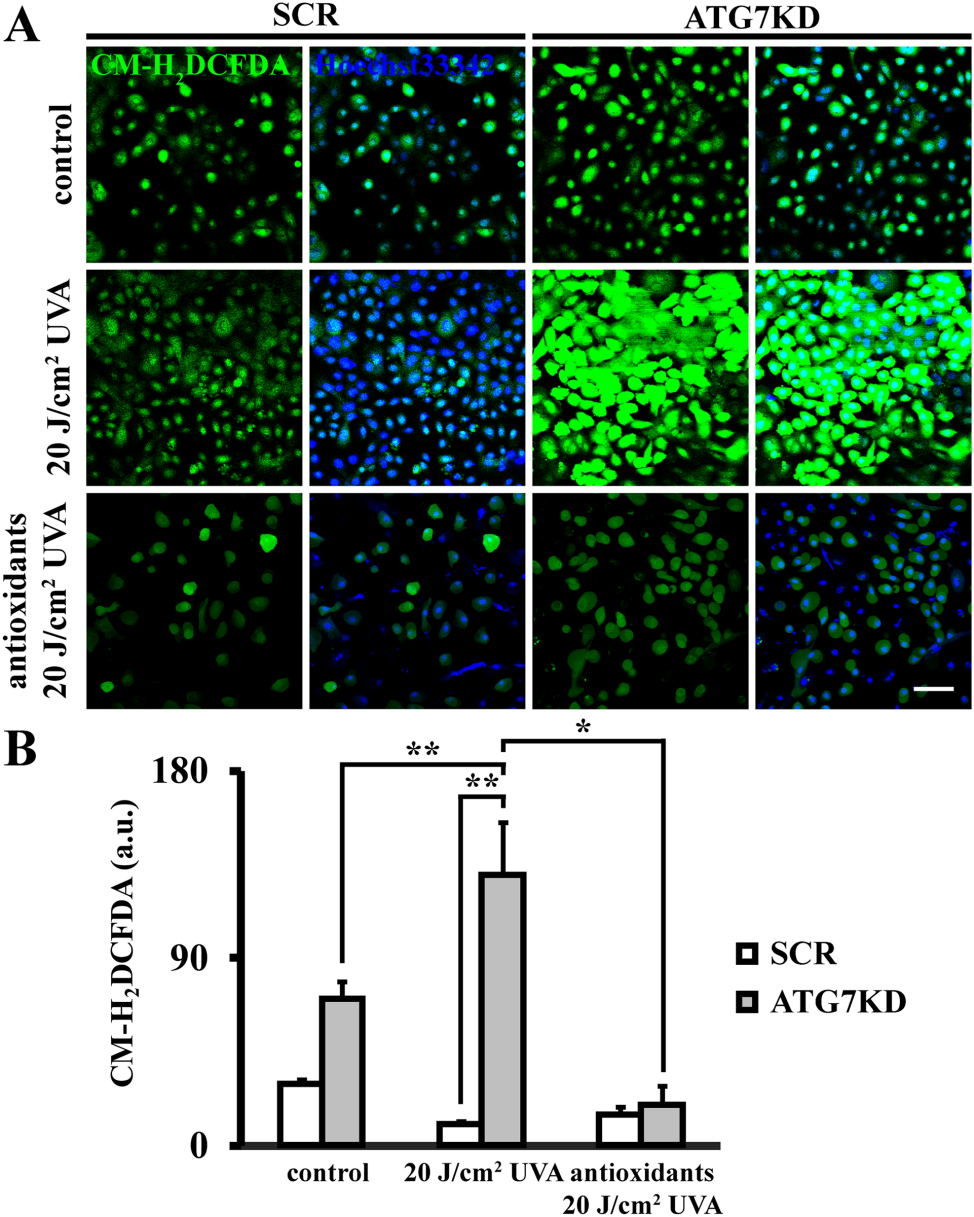
Autophagy regulates intracellular ROS levels in LSCs under UVA stress. (A) Intracellular ROS determined by CM-H_2_DCFDA staining of SCR and ATG7KD LSCs, with or without UVA and antioxidant pretreatment. Scale bar, 100 μm. (B) Quantification of ROS levels by mean fluorescence intensity. *p < .05, **p < .01.

Next, we sought to determine whether autophagy contributes to restore redox balance after UVA stress. To this end, intracellular ROS were evaluated by CM-H_2_DCFDA live imaging. After UVA irradiation, ROS remained at physiological level in SCR as well as antioxidant pre-treated LSCs compared to non-irradiated controls (10.5 ± 1.3 a.u. after UVA and 15.1 ± 3.5 a.u. with antioxidant pre-treatment compared to 29.5 ± 2.5 a.u. in controls, *p*>.05) (Fig 3). In contrast, ATG7KD LSCs showed a significant increase of intracellular ROS upon UVA exposure compared to non-irradiated counterparts (70.1 ± 9 a.u. in ATG7KD and 130.9 ± 25.4 a.u. after UVA *p* < .05) as well as irradiated, autophagy-competent LSCs (12.5-fold increase, *p* < .01). Conversely, treating ATG7KD LSCs with antioxidant mixture 16 hours prior to irradiation largely alleviated the accumulation of intracellular ROS in ATG7KD LSCs (19.8 ± 8.7 a.u. versus 130.9 ± 25.4 a.u. in UVA-treated ATG7KD LSC, *p* < .05). These data suggest a crucial role of autophagy in regulating redox balance in LSC’s stress response to UVA.

### Autophagy facilitates nucleocytoplasmic transport of PAX6 in response to UVA-induced ROS

**Fig 4:**
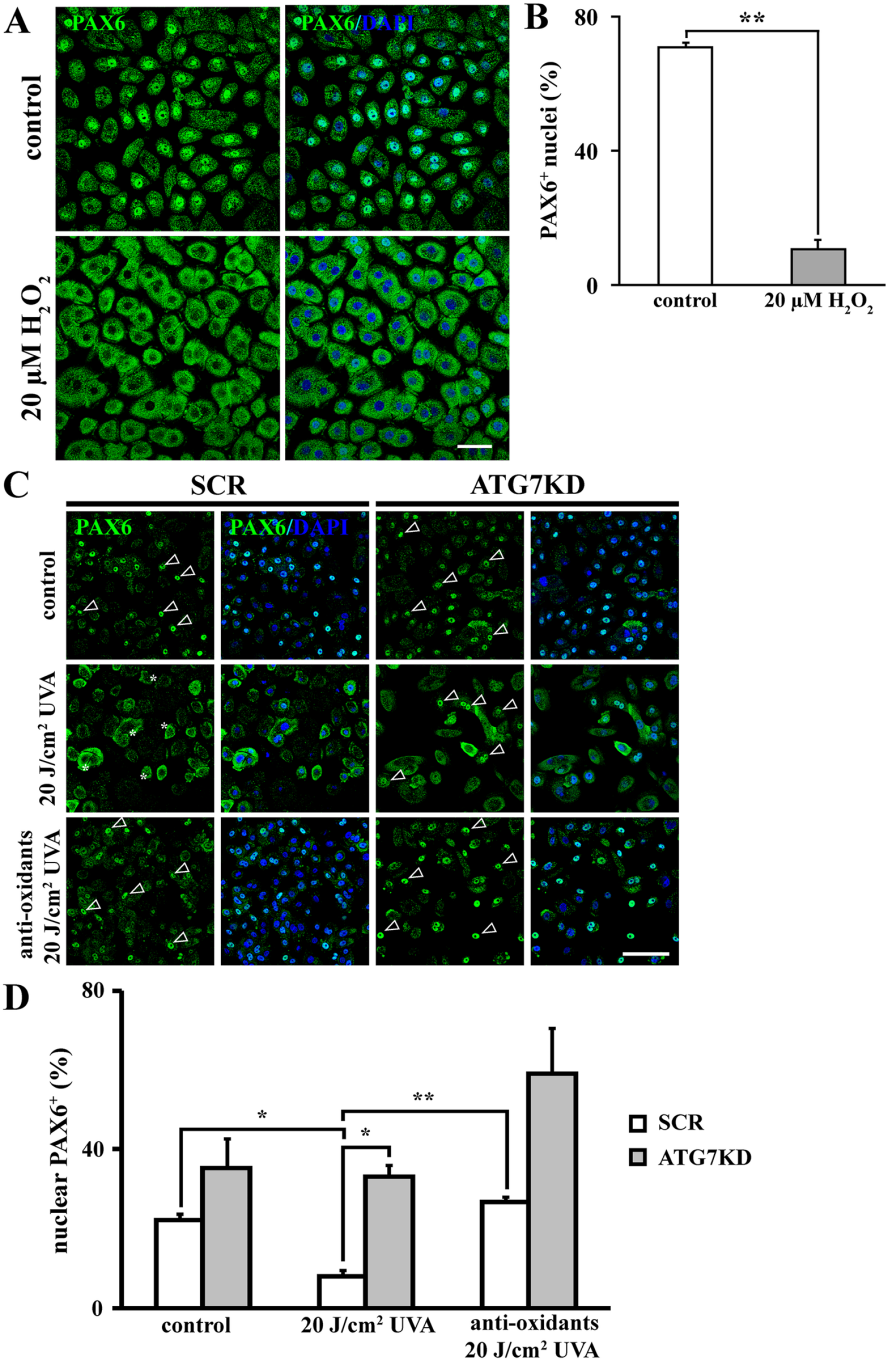
Autophagy mediates nuclear export of PAX6 in response to UVA-elicited oxidative stress. (A) Representative immunofluorescent images depicting subcellular localization of PAX6 in controls and H_2_O_2_-treated LSCs. Scale bar, 50 μm. (B) Quantification of cells expressing nuclear PAX6 after H_2_O_2_ treatment. (C) Representative micrographs of PAX6 subcellular localization in LSCs exposed to UVA with or without antioxidant pretreatment. Arrowheads indicate nuclear localization of PAX6, asterisks denote cytoplasmic distribution of PAX6. Scale bar, 100 μm. (D) Percentage of LSCs expressing nuclear PAX6. *p < .05, **p < .01.

To determine whether our model is suitable to study PAX6 response to oxidative stress, colonies were challenged with 20 μM H_2_O_2_ and subcellular expression of PAX6 was examined by immunofluorescence. Notably, LSC colonies displayed high levels of nuclear PAX6 expression on clonal day 12 (70.9 ± 1.5%) (Fig 4A & 4B). H_2_O_2_-induced oxidative stress resulted in nuclear depletion of PAX6 (10.8 ± 2.9% PAX6^+^ nuclei, *p* < .01 compared to controls), validating our *in vitro* ROS-responsive PAX6 model. Since we found that autophagy balances UVA-induced oxidative stress, we next asked whether PAX6 was differentially regulated in autophagy-competent and—deficient LSCs confronted with UVA-elicited ROS. To this end, the subcellular localization of PAX6 was studied in UVA-stressed SCR and ATG7KD LSCs by immunofluorescence. Interestingly, the percentage of LSCs expressing nuclear PAX6^+^ was significantly decreased in SCR LSC colonies after UVA exposure (8.0 ± 1.6%) compared to non-irradiated SCR LSCs (22.1 ± 1.8%, *p* < .01) (Fig 4C & 4D). In contrast, ATG7KD LSCs retained baseline levels of PAX6^+^ nuclei after irradiation (35.3 ± 7.5% in ATG7KD versus 33.2 ± 3.1% after UVA, *p*>.05). Of note, the reduction of nuclear PAX6^+^ cells in irradiated SCR LSCs correlated with the increase of cells with cytoplasmic PAX6 expression. In addition, antioxidant pre-treatment abolished the UVA-triggered PAX6 nuclear depletion observed in SCR LSCs evidenced by 26.6 ± 1.3% of nuclear PAX6^+^ cells in irradiated SCR LSCs (*p* < .01 compared to 8.0 ± 1.6% in irradiated LSCs). Collectively, data presented here suggest: (1) Nuclear-to-cytoplasmic protein transport, rather than transcriptional repression or acceleration of cytoplasmic PAX6 protein degradation, is the dominant mechanism by which UVA induces nuclear reduction of PAX6. (2) The UVA-induced PAX6 nucleocytoplasmic transportation is both ROS- and autophagy-dependent.

### UVA radiation induces cell cycle arrest in an autophagy-dependent, PAX6-mediated mechanism

**Fig 5:**
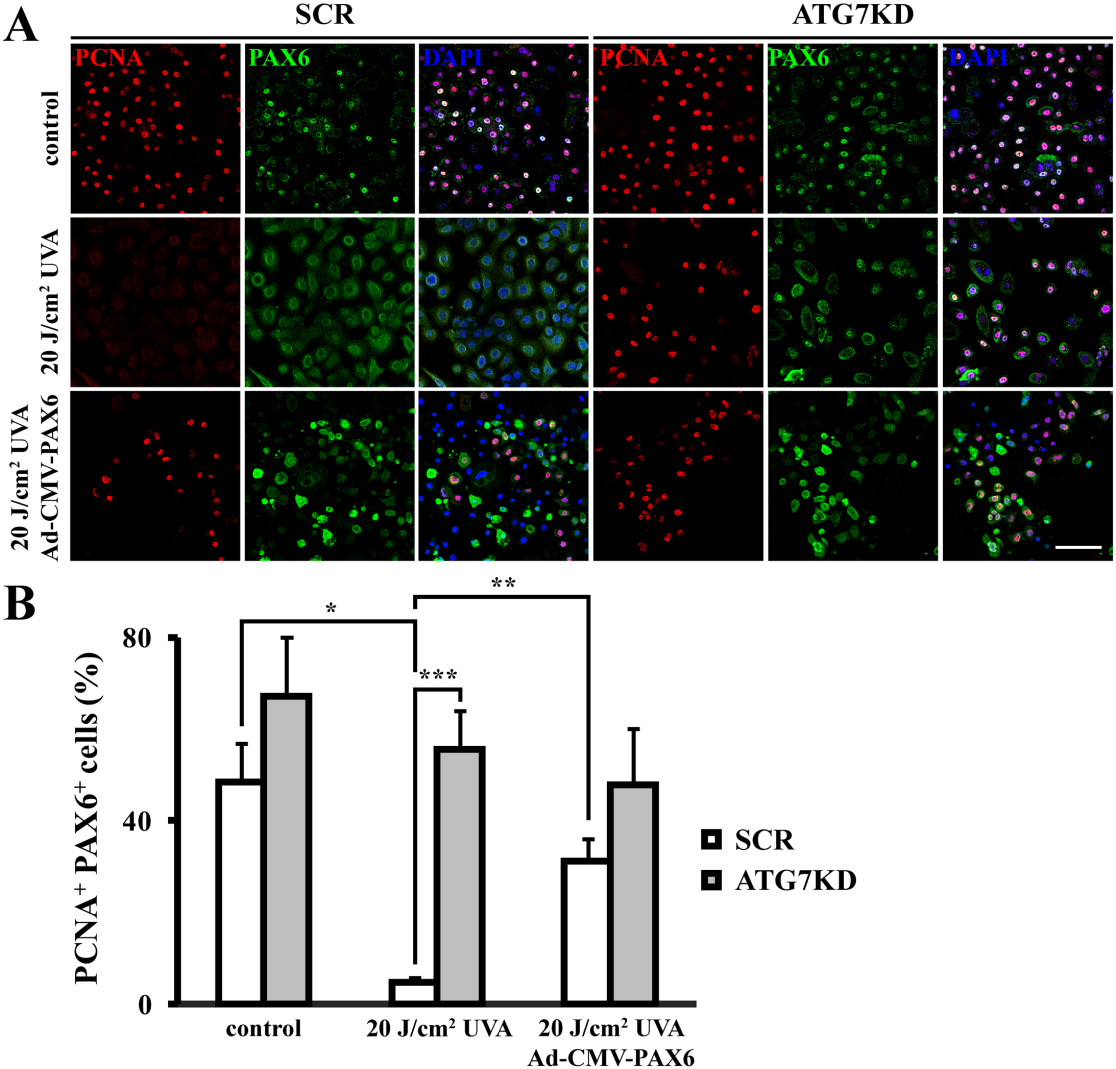
Autophagy mediates PCNA expression and PAX6 cyto-localization in UVA-irradiated LSC colonies. (A) Dual immunofluorescent staining against PCNA and PAX6 in UVA-irradiated LSC colonies with or without adenoviral overexpression of *PAX6*. Scale bar, 100 μm. (B) Quantification of PAX6^+^PCNA^+^ cells. *p < .05, **p < .01, ***p < .001.

Next, we aimed to determine whether nucleocytoplasmic translocation of PAX6 plays a role in cell cycle response during UVA stress. Therefore, we performed dual immunofluorescent staining against PAX6 and PCNA, a proliferative marker. Accompanying nuclear export of PAX6, UVA irradiation led to a significant reduction of PCNA expression in autophagy-competent LSC colonies from 52.8 ± 6.5% to 42.3 ± 4.0% PCNA^+^ PAX6^+^ cells (*p* < .01) (Fig 5), indicative of cell cycle arrest. Compared to irradiated counterparts, SCR LSCs pretreated with antioxidants retained proliferative activity and nuclear expression of PAX6 under UVA stress (32.2 ± 2.8% higher levels of PCNA^+^ PAX6^+^ cells compared to irradiated counterparts, *p* < .05). The number of PCNA^+^ PAX6^+^ cells remained high in ATG7KD LSCs following UVA irradiation (67.1 ± 12.9% and 76.7 ± 3.3% after UVA, *p*>.05). Interestingly, cells co-expressing PCNA and nuclear PAX6 after UVA exposure were significantly lower in SCR cells compared to ATG7KD LSCs under UVA stress (42.3 ± 4.0% in irradiated SCR and 76.7 ± 3.3% PCNA^+^ PAX6^+^ cells in irradiated ATG7KD, *p* < .01).

To determine whether reduced PCNA levels following UVA exposure results from nuclear depletion of PAX6, we restored the nuclear expression of PAX6 by adenoviral overexpression (S2 Fig). Compared to basal proliferation of unirradiated PAX6-expressing LSC colonies, adenoviral forced PAX6 expression in nuclear compartment clearly sustained the proliferative activity of LSC under UVA stress. The percentage of cycling PAX6^+^ PCNA^+^ cells was significantly lower in UVA-irradiated, Ad-CMV-null vector-infected LSCs (4.7 ± 1.1%) compared to Ad-CMV-null vector-infected controls (48.6 ± 8.3%, *p* < .05) (Fig 5 & S3 Fig). Intriguingly, overexpressing PAX6 abolished the UVA-induced downregulation of cycling cells (31.3 ± 4.8% in UVA-irradiated, Ad-CMV-PAX6 vector-infected LSC compared to 48.6 ± 8.3% in non-irradiated Ad-CMV-null vector-infected control LSCs, *p*>.05). Furthermore, the percentages of cycling PCNA^+^ PAX6^+^ cells in irradiated and non-irradiated Ad-CMV-null vector-infected ATG7KD LSCs were found similar to that in non-irradiated Ad-CMV-null vector-infected SCR LSCs (55.7 ± 8.4%, 67.1 ± 12.9%, and 48.6 ± 8.3%, *p*>.05). Lastly, adenoviral forced PAX6 expression in ATG7-KD LSCs did not correct the aberrant cell cycle progression under UVA stress, as the percentage of PAX6^+^ PCNA^+^ cells did not differ between non-irradiated Ad-CMV-null vector-infected ATG7KD, UVA-irradiated Ad-CMV-null vector-infected ATG7KD and UVA-irradiated Ad-CMV-PAX6 vector-infected ATG7KD (67.1 ± 12.9%, 55.7 ± 8.4% and 48.9 ± 12.1%, *p*>.05).

**Fig 6:**
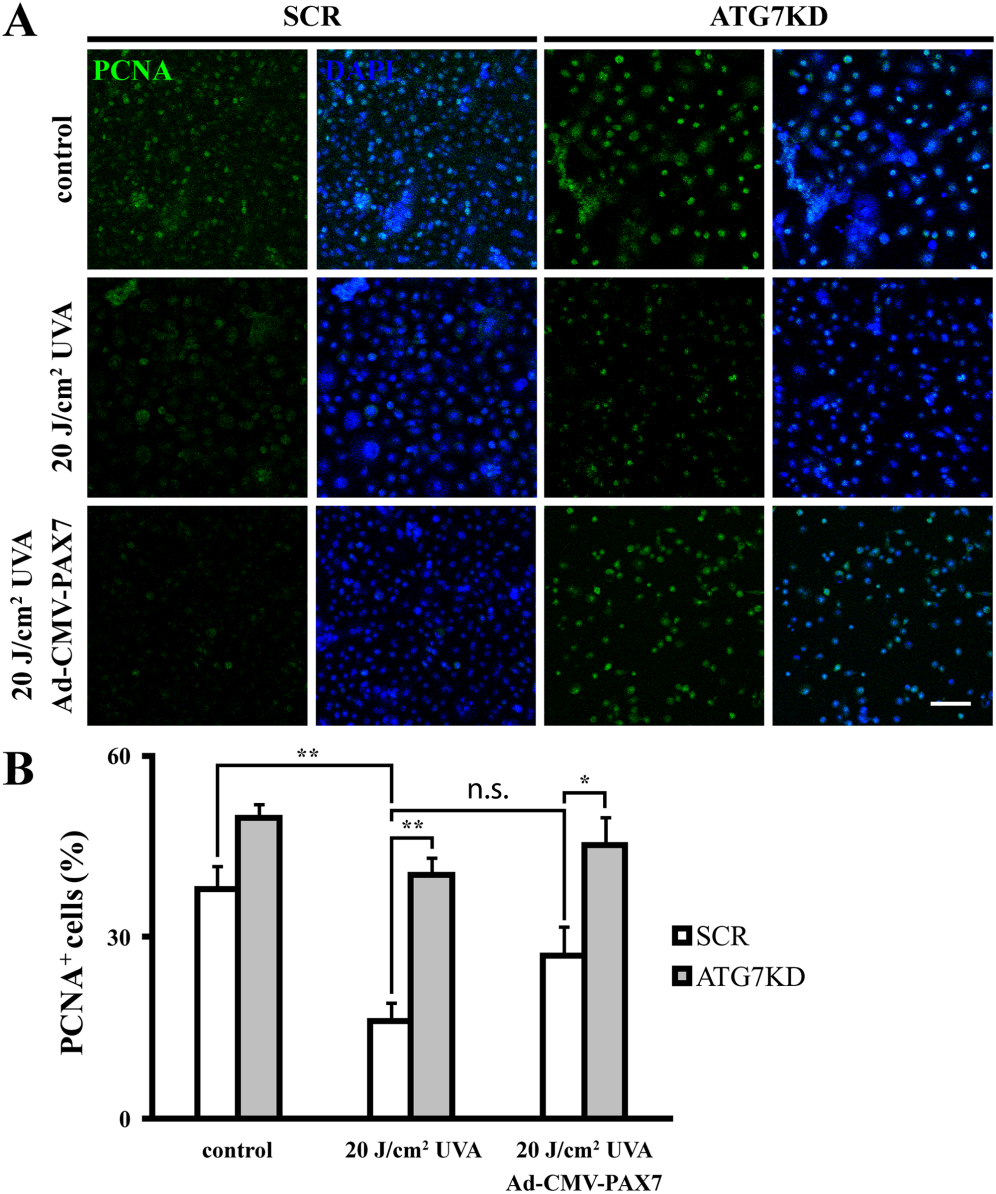
PAX7 overexpression in nuclear PAX6-depleted LSCs does not rescue UVA-induced PCNA downregulation. (A) Representative micrographs of PCNA immunofluorescence in irradiated SCR and ATG7KD colonies with or without adenoviral overexpression of PAX7. Scale bar, 100 μm. (B) Quantitative analysis of nuclear PCNA expression. *p < .05, **p < .01.

To determine whether the UVA-induced PCNA downregulation in LSCs is a gene function unique to ocular tissue-resident PAX6, we ectopically overexpressed *PAX7*, a PAX family member normally absent from ocular tissue. qPCR analysis was used to determine *PAX7* expression in Ad-CMV-PAX7-infected and control LSC colonies 72 hours after viral transduction. *PAX6* mRNA was found highly expressed in Ad-CMV-null controls and not affected by adenoviral forced *PAX7* overexpression (ΔCt = 2.36 ± 0.36, *GAPDH* Ct = 23.00 ± 0.34 in Ad-CMV-null versus ΔCt = 2.66 ± 0.33, *GAPDH* Ct = 22.41 ± 0.27 in Ad-CMV-PAX7, 40 thermal cycles, *p*>.05). In contrast, *PAX7* was undetectable in Ad-CMV-null controls, while *PAX7* expression was found significantly increased in colonies transfected with Ad-CMV-PAX7 (ΔCt = 0.21 ± 0.17, *GAPDH* Ct = 22.41 ± 0.27), confirming *PAX7* overexpression. To determine the effect of ectopic PAX7 on cell cycle regulation, PCNA expression was assessed by immunofluorescence in Ad-CMV-PAX7 LSCs. As expected, the number of PCNA-expressing cells was found significantly reduced by irradiation in scrambled KD and control vector-transduced LSCs (38.01 ± 3.73% in non-irradiated versus 16.12 ± 3.02% in irradiated SCR, Ad-CMV-null, *p* < .01) (Fig 6). In contrast to adenoviral PAX6 restoration of PCNA expression in UVA-stressed LSCs (Fig 5), PCNA expression was not rescued by adenoviral-forced PAX7 expression in irradiated, nuclear PAX6-depleted LSCs (26.91 ±4.8% PCNA^+^ cells, *p*>.05). Taken together, these results suggest a PAX6-specific mediation on UVA-induced PCNA downregulation.

**Fig 7:**
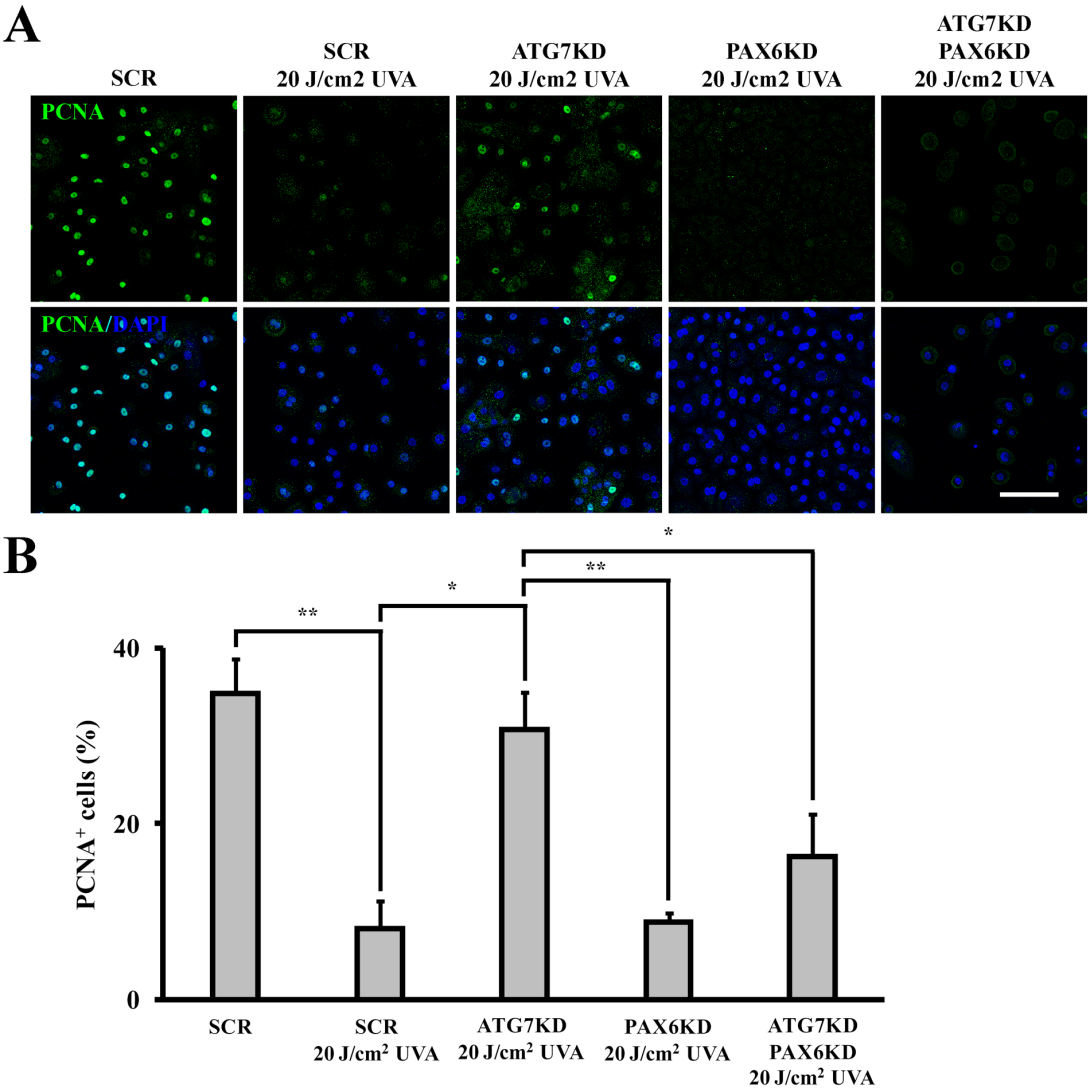
PAX6KD attenuates the uncurbed cell cycle response in ATG7KD LSCs following UVA irradiation. (A) Representative images of PCNA immunofluorescence in ATG7KD, PAX6KD and ATG7/PAX6 KD colonies following UVA irradiation. Scale bar, 100 μm. (B) Quantification of cells expressing PCNA in ATG7KD, PAX6KD or double KD LSCs. *p < .05, **p < .01.

To verify autophagy-dependent PAX6’s regulatory effects on LSC’s clonal proliferation, we knocked down PAX6 by siRNA (97% of KD efficiency, S4 Fig). A significantly lower percentage of proliferative cells was found in UVA-irradiated SCR LSCs (34.7 ± 4% in non-irradiated SCR controls versus 7.9 ± 3.3% in UVA-irradiated SCR LSCs, *p* < .01) (Fig 7). While the number of PCNA^+^ cells in ATG7KD LSCs was not reduced by UVA irradiation (30.6 ± 4.4% in irradiated ATG7KD versus 34.7 ± 4% in non-irradiated ATG7KD, *p*>.05), PAX6KD LSCs displayed equally low levels of PCNA^+^ proliferative cells as irradiated SCR LSCs (8.85 ± 0.9 PCNA^+^ cells, *p*>.05). Importantly, concomitant knockdown of both ATG7 and PAX6 restored the UVA-induced cycle arrest response in irradiated LSCs, as the percentage of PCNA^+^ cells in irradiated ATG7/PAX6 double KD LSCs was reduced to a level of only 16.2 ± 4.9%, significantly lower than those observed in non-irradiated SCR LSC (30.6 ± 4.4%, *p* < .05) and irradiated ATG7KD LSC (30.6 ± 4.4%, *p* < .05). Taken together, our data suggest PAX6 functions as a unique molecular intermediate downstream to ATG7-mediated autophagy to regulate the cell cycle kinetics during LSC’s ultraviolet stress response.

### UVA-induced, autophagy-mediated nuclear depletion of PAX6 arrests cell cycle by activating cell cycle inhibitor p21

**Fig 8:**
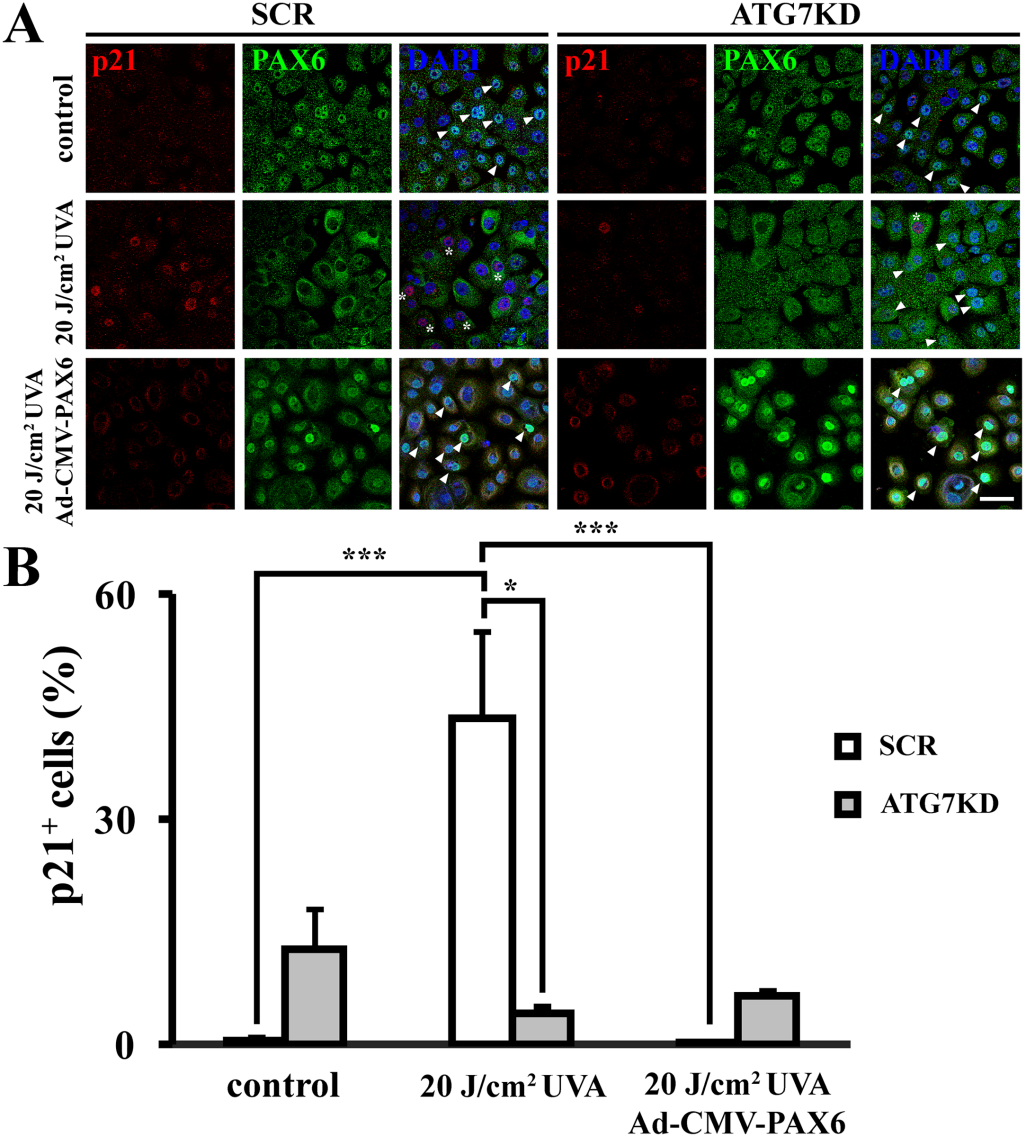
Autophagy mediates p21 induction in response to UVA via nuclear export of PAX6. (A) Micrographs of p21 and PAX6 cyto-localization in irradiated SCR and ATG7KD LSCs with or without PAX6 overexpression. Arrowheads denote PAX6^+^ p21^-^ nuclei, asterisks indicate cells expressing nuclear p21 and cytoplasmic PAX6. Scale bar, 50 μm. (B) Quantification of p21^+^ nuclei. *p < .05, ***p < .001.

To further dissect the mechanism by which autophagy induces cell cycle arrest in response to UVA, we investigated nuclear expression of p21, a negative regulator for cell cycle progression. Following UVA irradiation, autophagy-competent LSCs upregulated p21 expression from 0.6 ± 0.3% to 43.5 ± 11.6% compared to non-irradiated controls (*p* < .01) (Fig 8 & S5 Fig). Forced PAX6 expression abolished the UVA-induced p21 expression in SCR colonies (0.25 ± 0.2% compared to 43.5 ± 11.6% in irradiated SCR controls, *p* < .05). In contrast, UVA radiation failed to induce p21 expression in autophagy-deficient LSCs (12.6 ± 5.4% p21^+^ in non-irradiated versus 4.2 ± 1.2% after irradiation, *p*>.05). Moreover, p21 expression was not induced by UVA irradiation in PAX6-overexpressing ATG7KD colonies (6.5 ± 0.9% p21^+^ cells) compared to irradiated Ad-CMV-null controls (4.2 ± 1.2% p21^+^ cells, *p*>.05).

**Fig 9:**
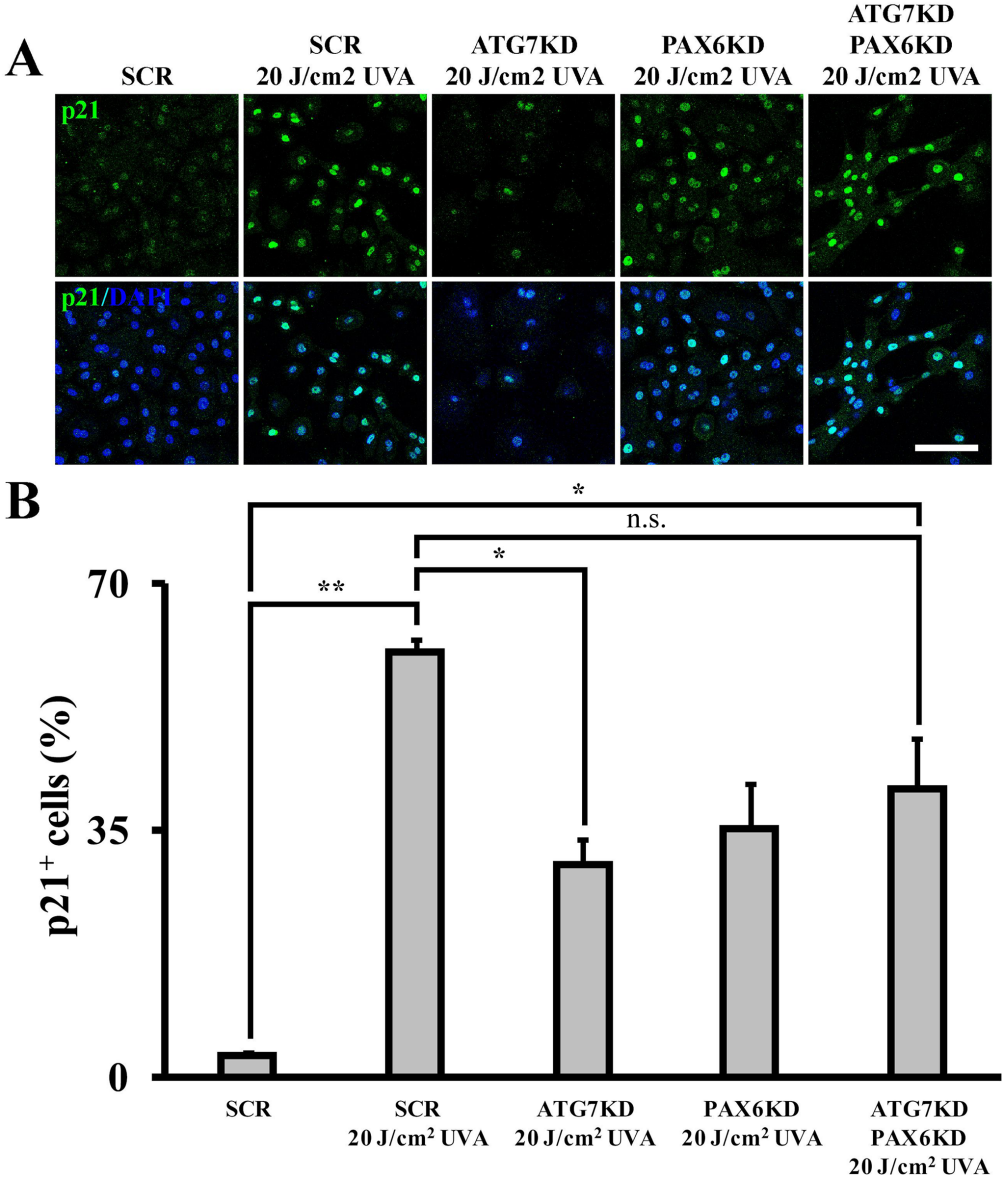
PAX6KD restores UVA-induced, p21-mediated cell cycle arrest in ATG7KD LSC. (A) Immunofluorescence of p21 in LSC colonies with KD of ATG7, PAX6 or both. Scale bar, 100 μm. (B) Quantification of p21-expressing cells in ATG7KD, PAX6KD or ATG7/PAX6 double KD LSCs. *p < .05, **p < .01.

To attest whether p21 expression is repressed by nuclear PAX6 in an autophagy-dependent mechanism, we examined p21 expression in UVA-irradiated ATG7KD, PAX6KD, and ATG7/PAX6 double KD LSCs. While p21 was significantly induced by UVA irradiation in SCR LSCs (60.3 ± 0.5% p21^+^ cells in the UVA group versus 3.1 ± 0.5% in non-irradiated controls, *p* < .01), p21 induction was not observed in UVA-irradiated ATG7KD (30.1 ± 3.8%) (Fig 9). Notably, the UVA-induced p21 expression was restored by knocking down PAX6 in ATG7KD LSCs (41.1 ± 7%). Collectively, these data demonstrate that the autophagy induces cell cycle arrest in UVA-irradiated LSCs by unlocking PAX6-repressed p21 expression.

## Discussion

**Fig 10:**
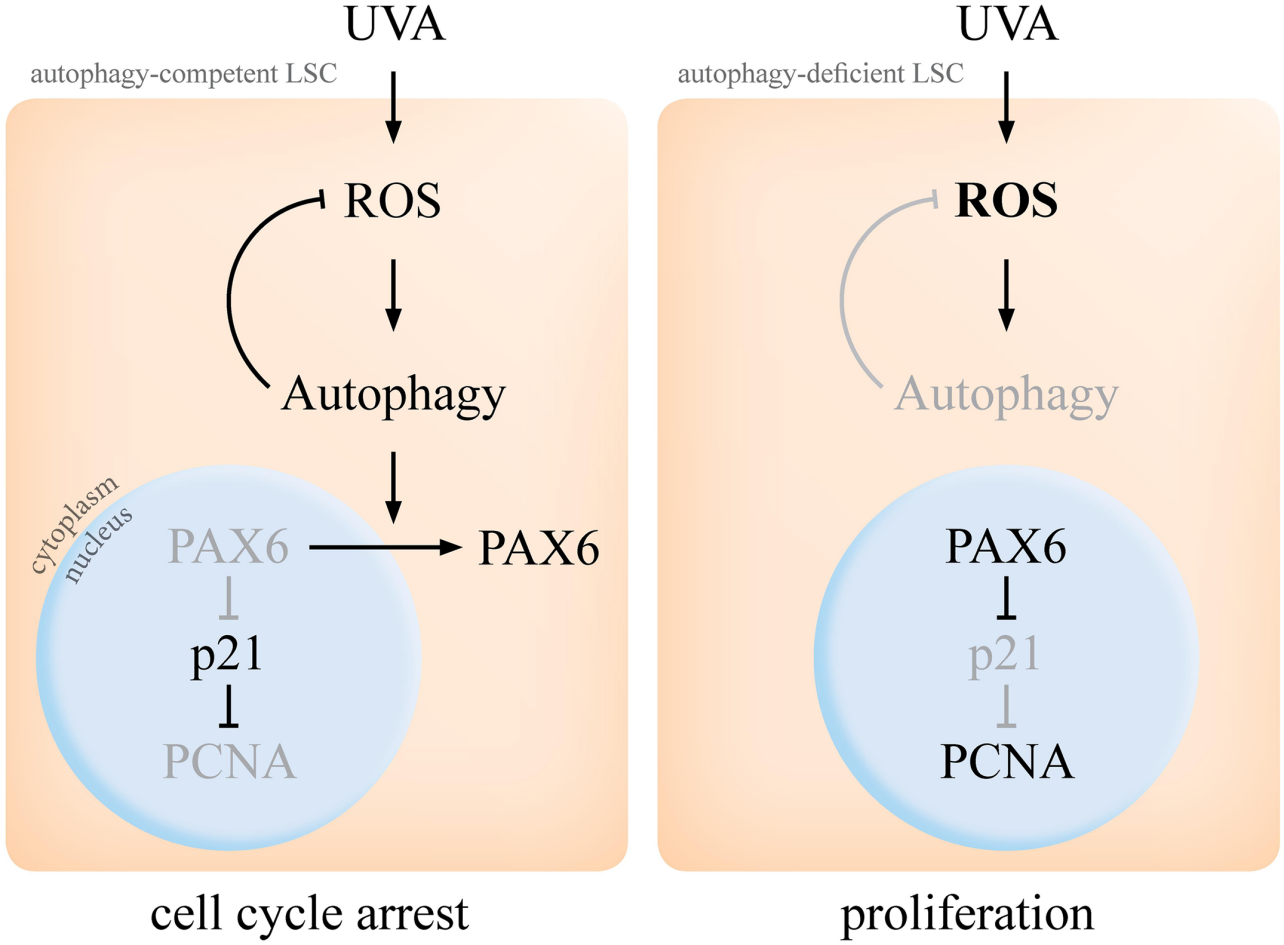
Model of autophagy-mediated regulation of PAX6 cyto-localization and cell cycle in UVA-stressed LSCs. In autophagy-competent LSCs, UVA activates autophagy by inducing ROS. In return, autophagic activity stabilizes intracellular ROS at physiological level. Furthermore, autophagy regulates cell cycle by facilitating cytoplasmic export of nuclear PAX6, which normally represses p21 in PCNA^+^, proliferative LSCs. In lack of functional autophagy, UVA induces excess levels of ROS, PAX6 is retained in the nucleus and the p21-mediated cell cycle response is hampered.

In current study, we identified autophagy as an UV stress sensor in LSCs. Dual functional roles for autophagy in cellular stress response were further suggested by alteration of redox status and cell cycle progression in UVA-irradiated LSC. Activation of autophagy in LSC contributes to restoration of intracellular redox balance and adaptive cell cycle response. We propose a novel PAX6-p21 molecular mechanism underpinning the autophagy-mediated stress response (Fig 10).

Autophagosome accumulation has been reported in the UV stress response of epidermal keratinocytes [21], whether autophagic flux is increased or decreased remains undetermined. Increased number of autophagosomes in stressed cells may result from elevated autophagic flux or impaired lysosomal degradation of autophagosomes. Our autophagic flux experiment suggests the autophagosome accumulation observed in UVA-irradiated LSCs, corneal keratinocyte progenitors, resulted from increased autophagic flux.

Zhao *et al*. show that autophagy exerts a cytoprotective effect by selective degradation of UV-oxidized macromolecules [21]. Here, we identified autophagy as an alternative antioxidant mechanism in LSCs to reduce ROS elicited by UVA (Fig 3). Whether the autophagy-mediated redox balance contributes to reduce UVA-induced oxidative damage in LSCs requires further investigation. While it is known that high dose irradiation induces cellular apoptosis, physiological effects of low dose UVA radiation on LSCs remains unclear. Using a non-lethal dose of UVA irradiation, we found that stressed LSCs employ autophagy as a molecular machinery to curb cell cycle (Figs 5 & 8). Interestingly, low level of ROS was identified as a key signaling event in the autophagy-mediated cell cycle response, since antioxidant pretreatment was found to abrogate the UVA-induced cell cycle arrest. Impairing autophagic machinery by ATG7KD resulted in excessive ROS accumulation and failure of cell cycle response in UVA-stressed LSCs. Paradoxically, high level of ROS in autophagy-deficient LSCs failed to induce cell cycle arrest (Figs 3, 5 & 8). Therefore, ATG7-mediated autophagy is intrinsic to redox balance and cell cycle response in LSC’s stress response to mitigate UVA-elicited oxidative stress.

PAX6 deregulation and oxidative stress have been identified as early pathogenic events in various ocular surface diseases [17,26–29]. A direct molecular interaction between PAX6 and ROS is suggested by rapid nucleocytoplasmic translocation of PAX6 in hydrogen peroxide-treated corneal cells [19]. Data presented in current study further suggest a functional role for the ROS-driven PAX6 mobilization in stressed corneal cells, *i*.*e*. mediation of cell cycle response. In our experiment, PAX6 and PCNA were found mostly co-localized in the nuclear compartment of proliferative LSCs in unstressed conditions (Fig 5). In contrast to the cytostatic growth effect in differentiated corneal cell [30], nuclear PAX6 at physiological dose in non-irradiated clonal cells might have a stimulatory effect in cell cycling. Following cytoplasmic relocation of PAX6, we observed a cell cycle arrest via p21-mediated PCNA downregulation (Figs 8 & 9). PAX6 is known to differentially regulate cell cycle via various mechanisms. For instance, PAX6 expression has been associated with proliferation and cell cycle progression of colorectal cancer cells, non-small cell lung carcinoma cells and breast cancer cells [31–33]. A mechanistic for the PAX6-driven cell cycle progression is recently suggested by a recent work of Li *et al*. [34]. In their work, Li and colleagues reported that lentiviral overexpression of PAX6 in retinoblastoma cells results in downregulation of p21 and impairs the p53-mediated cell cycle arrest response through reducing p53-p21 molecular interaction. Although the effect of PAX6 on cell cycle progression observed in their study is in line with ours, the underlying mechanism for PAX6-driven cellular proliferation might differ. As a mutually exclusive expression of PAX6 and p21 in the nuclear compartment was observed during LSC’s stress response in our work (Fig 8), we postulate de-repression of cell cycle inhibitor p21 by nuclear export of PAX6 as the mechanism for UVA-induced cell cycle arrest. In contrast, PAX6 downregulation was associated with cell cycle progression and proliferation of corneal epithelial cells in PAX6^+/-^ corneal epithelia [35]. Genetic depletion of *Pax6* in murine neuroepithelium led to clonal expansion of progenitor pool by downregulating p21 [36]. Li *et al*. showed that EGF induced cellular proliferation via PAX6 downregulation [37]. Furthermore, Ouyang *et al*. demonstrated that PAX6 overexpression induced cell cycle exit in proliferative corneal epithelial cells [30]. The differential cell cycle regulation between our study and the others suggest a stress context-dependent PAX6 function.

Recent epidemiological studies suggest associations of solar UV radiation and PAX6 deregulation in ocular surface diseases [26,38–42]. Our data suggest that PAX6 mediates LSC’s UVA stress response by regulating cell cycle. Although the functional significance of cell cycle arrest via nucleocytoplasmic PAX6 relocation in LSC’s stress response remains unclear, we surmise that pausing cell cycle might contribute to stem cell’s self-repair and autophagic clearance of UV-induced cellular damage. Interestingly, PAX7, a PAX family member expressed in muscle precursor cells, directs *in ovo* myogenesis by regulating proliferation of UV-stimulated myoblasts [43,44]. To attest whether ocular-resident PAX genes, *i*.*e*. PAX6, regulate UVA cell cycle response of LSCs, we expressed *PAX7* in nuclear PAX6-depleted LSCs in UVA stress context. In contrast to adenoviral PAX6 overexpression experiments, forced PAX7 expression did not affect autophagy-mediated UVA cell cycle response (Fig 6). Based on these findings, we conclude that the “UVA-autophagy-PAX-cell cycle” axis is specific to PAX6 in ocular stem cells. While it is surmised that PAX7 cannot replace PAX6 to orchestrate complex ocular transcriptional network, the detailed mechanism for PAX6-specific cell cycle regulation in UVA-stressed LSCs warrants further studies.

It is known that cells employ master transcription factors, such as p53 and Nrf2, to orchestrate stress responses, including apoptosis, cell cycle regulation and antioxidant defense to repair oxidative damage. The transcriptional activity of stress response transcription factors is controlled by dynamic subcellular localization with autophagy lately identified as a new regulatory mechanism [45–47]. Two mechanisms by which ROS drive PAX6 nuclear-to-cytoplasmic trafficking are oxidative modification of its nuclear export signal and oxidation of its adaptor proteins [48,49]. In the current study, we showed that low dose of ROS in autophagy-competent LSC induced cytoplasmic relocation of PAX6, while excessive levels of ROS in autophagy-deficient LSCs failed to drive the relocation (Figs 3 & 4). These findings clearly indicate an autophagic regulation of PAX6’ ROS response. Although physiological protein turnover of PAX6 mainly depends on proteosomal degradation [50], we do not rule out the potential of synergistic (or alternative) autophagic clearance of PAX6 in UVA stress context. Autophagy was reported to navigate intracellular trafficking of PAX6 by indirectly degrading PAX6 adaptor proteins, such as TRIM44 and SPARC [51–54]. Whether or not autophagy responds to ROS stress by selectively targeting PAX6 adaptor proteins to regulate cell cycle response requires further investigations.

In conclusion, our data indicate that autophagy mediates cell cycle response of LSCs following UVA exposure via a novel PAX6-p21 molecular cascade. The spatial dynamics of PAX6 recruitment to nucleocytoplasmic compartments represent a novel post-translational regulation for autophagy-mediated oxidative stress response. Functional enhancement of autophagy might thus represent a novel therapeutic strategy in treating UVA-associated, PAX6-deregulated ocular surface degenerative diseases.

## Supporting information

S1 Fig Non-lethal UVA irradiance to SCR and ATG7KD LSCs. Percentage of apoptotic cells assessed by TUNEL staining in SCR and ATG7KD following low dose UVA irradiation of 20 J/cm^2^.
(TIF)

S2 Fig Adenoviral-assisted overexpression of PAX6 in human LSC colonies. (A) Western blot of PAX6 expression in Ad-CMV-null and Ad-CMV-PAX6 infected cells. (B) Densitometry of PAX6 expression normalized to GAPDH levels. **p < .01 of 3 independent experiments.
(TIF)

S3 Fig PAX6/PCNA co-localization in irradiated ATG7KD colonies with adenoviral PAX6 overexpression. (A) Stacked bar diagram and (B) statistics of PAX6^+^PCNA^-^, PAX6^+^PCNA^+^, PAX6^-^PCNA^+^, and PAX6^-^PCNA^-^ cells. *p < .05 compared to SCR, Ad-CMV-null controls, ^#^*p* < .05 compared to SCR, Ad-CMV-null, UVA-irradiated counterparts.
(TIF)

S4 Fig Efficiency of siRNA-mediated KD of PAX6 in LSC colonies. (A) PAX6 immunofluorescence in SCR and PAX6KD LSC colonies. Scale bar, 100 μm. (B) Mean fluorescence intensity of PAX6 in SCR and PAX6KD LSCs. ***p < .001.
(TIF)

S5 Fig PAX6/PCNA co-localization in irradiated ATG7KD, PAX6-overexpressing LSC colonies. (A) Stacked bar diagram and (B) statistics of percentages of PAX6^+^p21^-^, PAX6^+^p21^+^, PAX6^-^p21^+^, and PAX6^-^p21^-^ cells. *p < .05 compared to SCR, Ad-CMV-null controls, ^#^*p* < .05 compared to SCR, Ad-CMV-null, UVA-irradiated counterparts.
(TIF)
