# Supplementary figures and images for: Autophagy mediates cell cycle response by regulating nucleocytoplasmic transport of PAX6 in limbal stem cells under ultraviolet-A stress
Source: PLoS One. 2017 Jul 10;12(7):e0180868. doi: 10.1371/journal.pone.0180868 (PMC5507275; doi:10.1371/journal.pone.0180868)

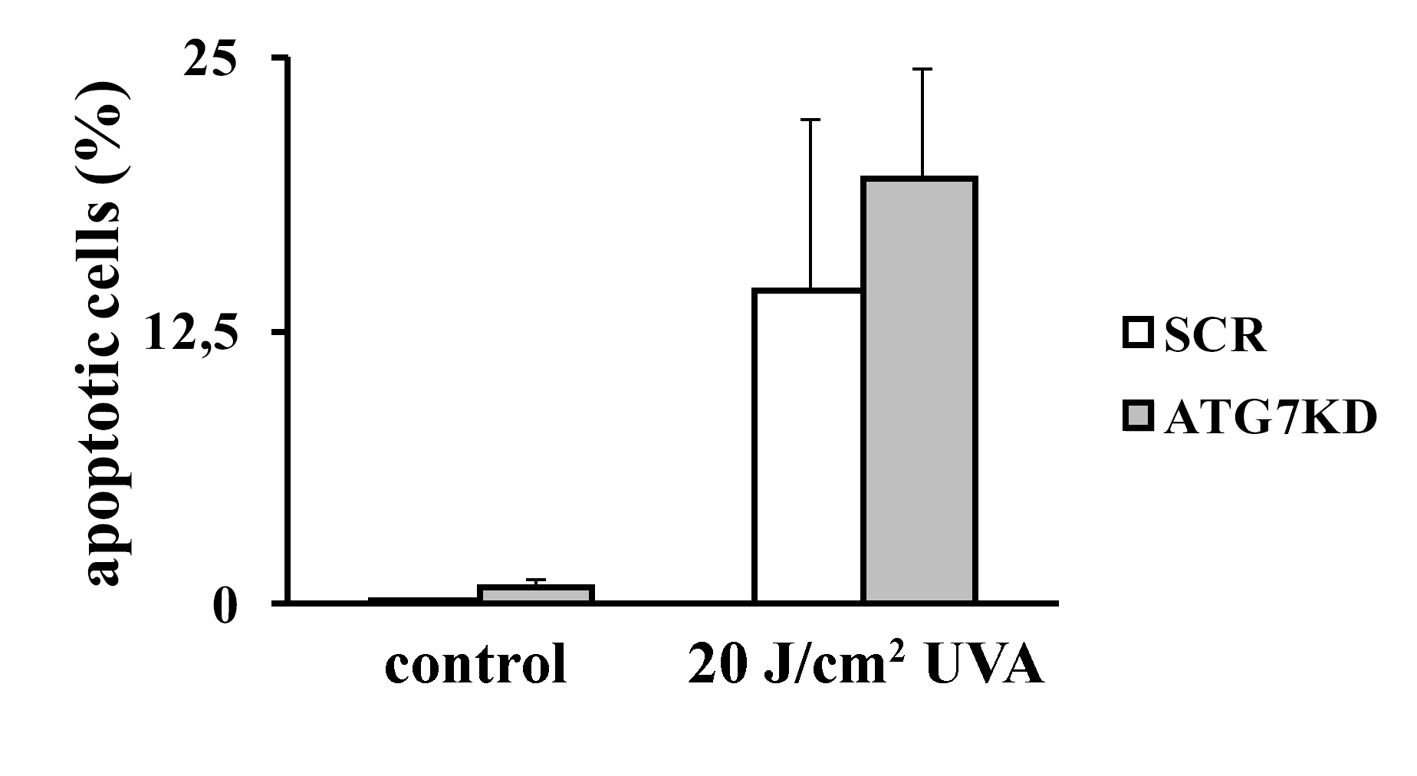

Supplement: S1 Fig — Percentage of apoptotic cells assessed by TUNEL staining in SCR and ATG7KD following low dose UVA irradiation of 20 J/cm2. (TIF) [file pone.0180868.s001.tif]

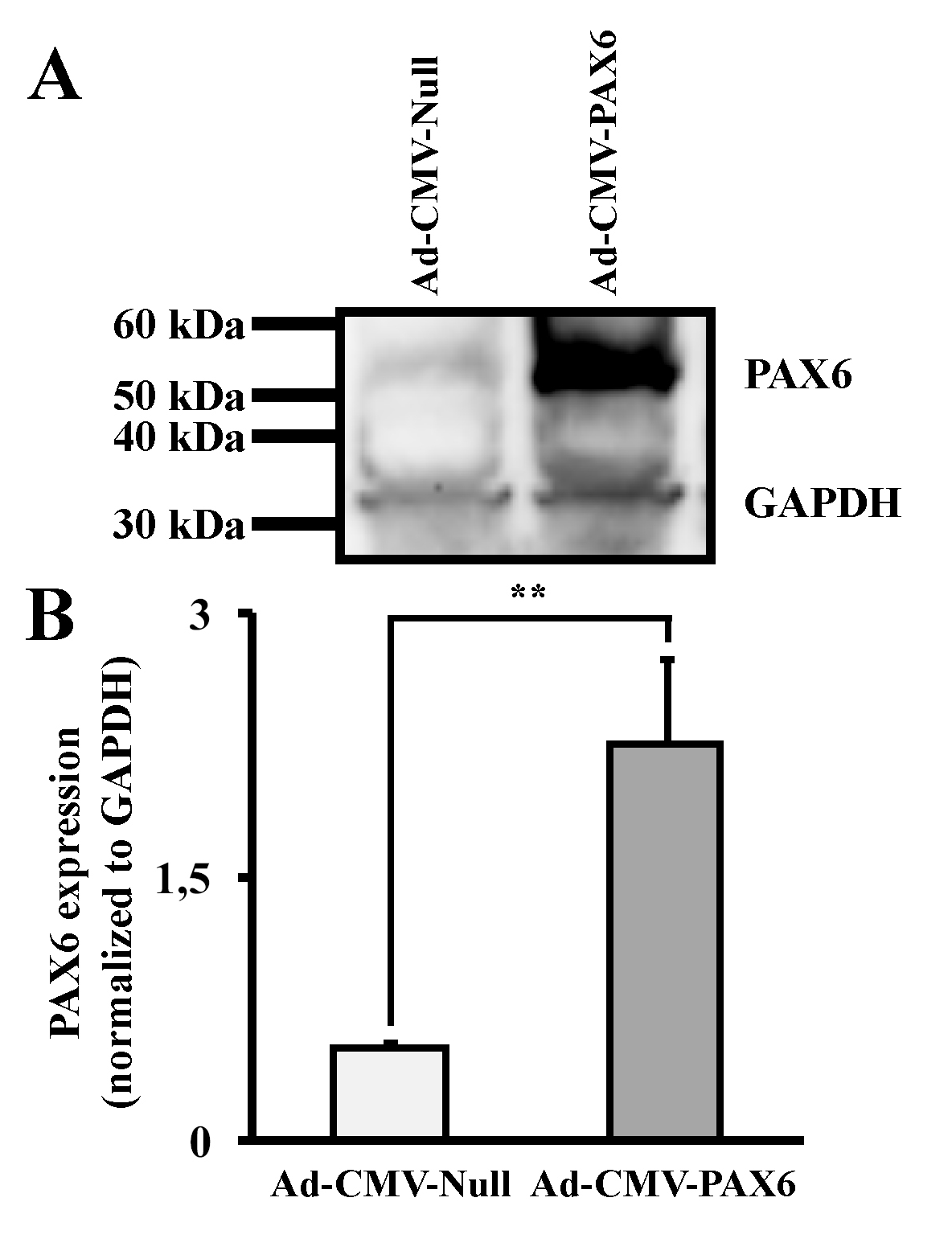

Supplement: S2 Fig — (A) Western blot of PAX6 expression in Ad-CMV-null and Ad-CMV-PAX6 infected cells. (B) Densitometry of PAX6 expression normalized to GAPDH levels. **p < .01 of 3 independent experiments. (TIF) [file pone.0180868.s002.tif]

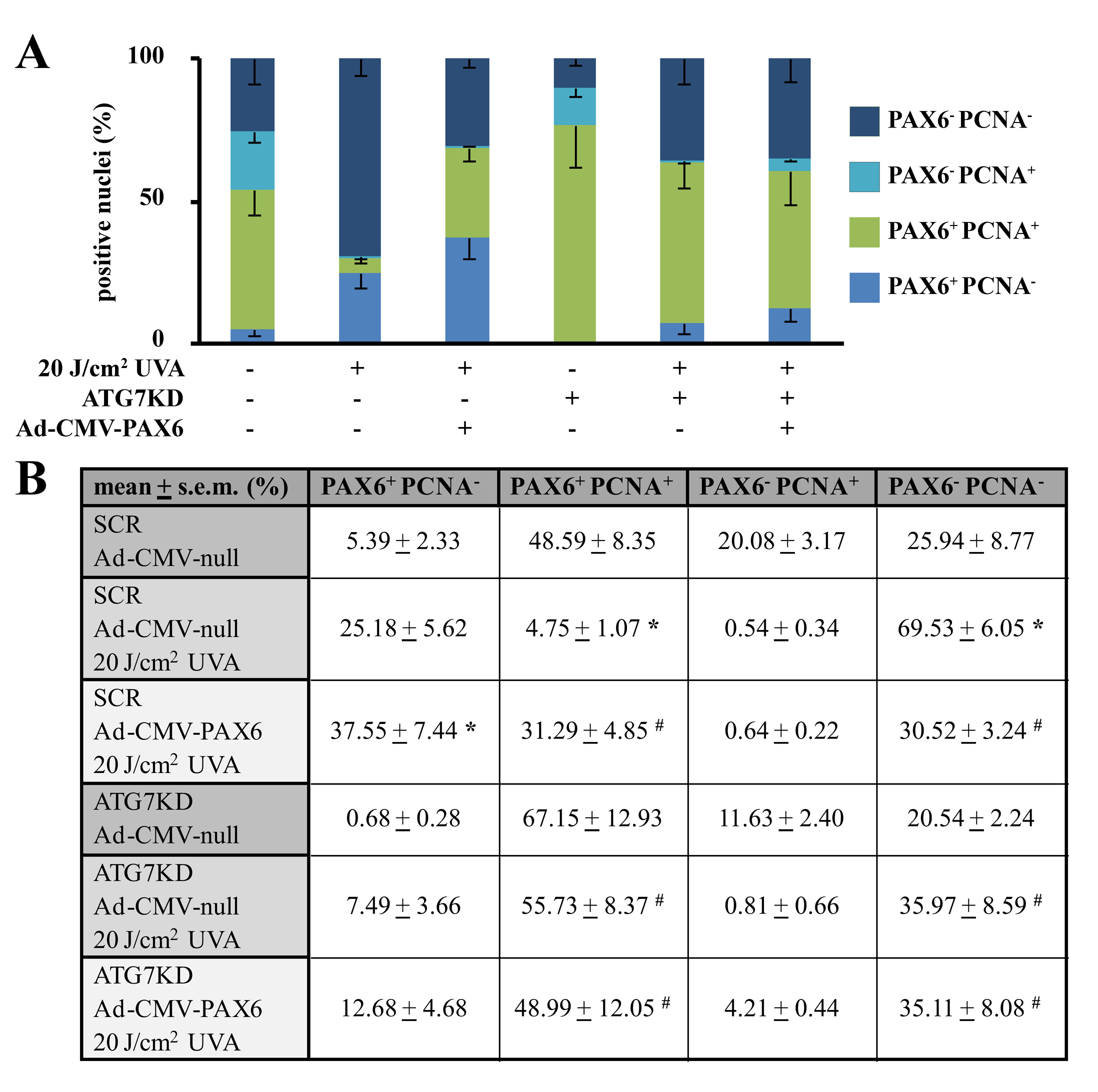

Supplement: S3 Fig — (A) Stacked bar diagram and (B) statistics of PAX6+PCNA-, PAX6+PCNA+, PAX6-PCNA+, and PAX6-PCNA- cells. *p < .05 compared to SCR, Ad-CMV-null controls, #p < .05 compared to SCR, Ad-CMV-null, UVA-irradiated counterparts. (TIF) [file pone.0180868.s003.tif]

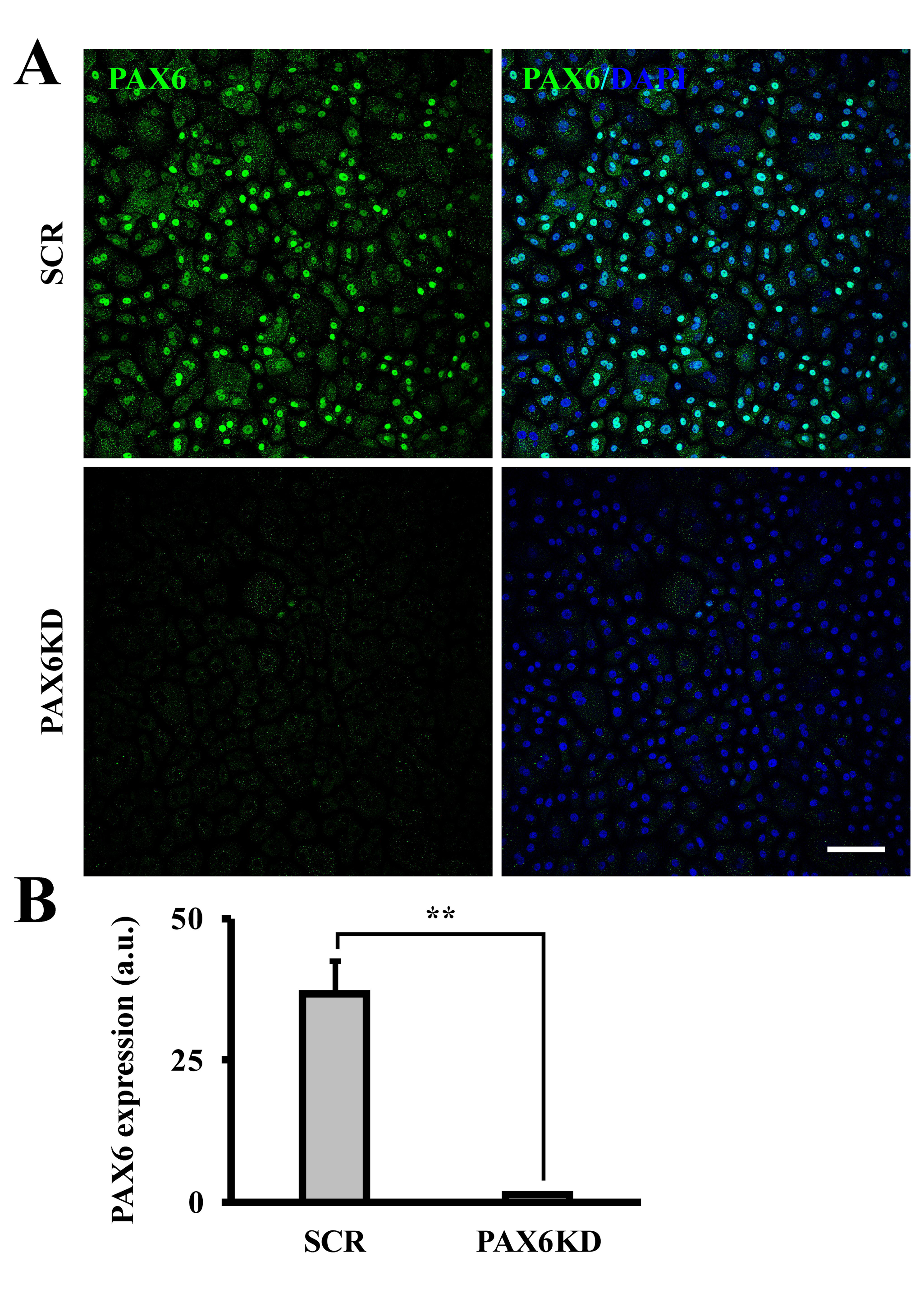

Supplement: S4 Fig — (A) PAX6 immunofluorescence in SCR and PAX6KD LSC colonies. Scale bar, 100 μm. (B) Mean fluorescence intensity of PAX6 in SCR and PAX6KD LSCs. ***p < .001. (TIF) [file pone.0180868.s004.tif]

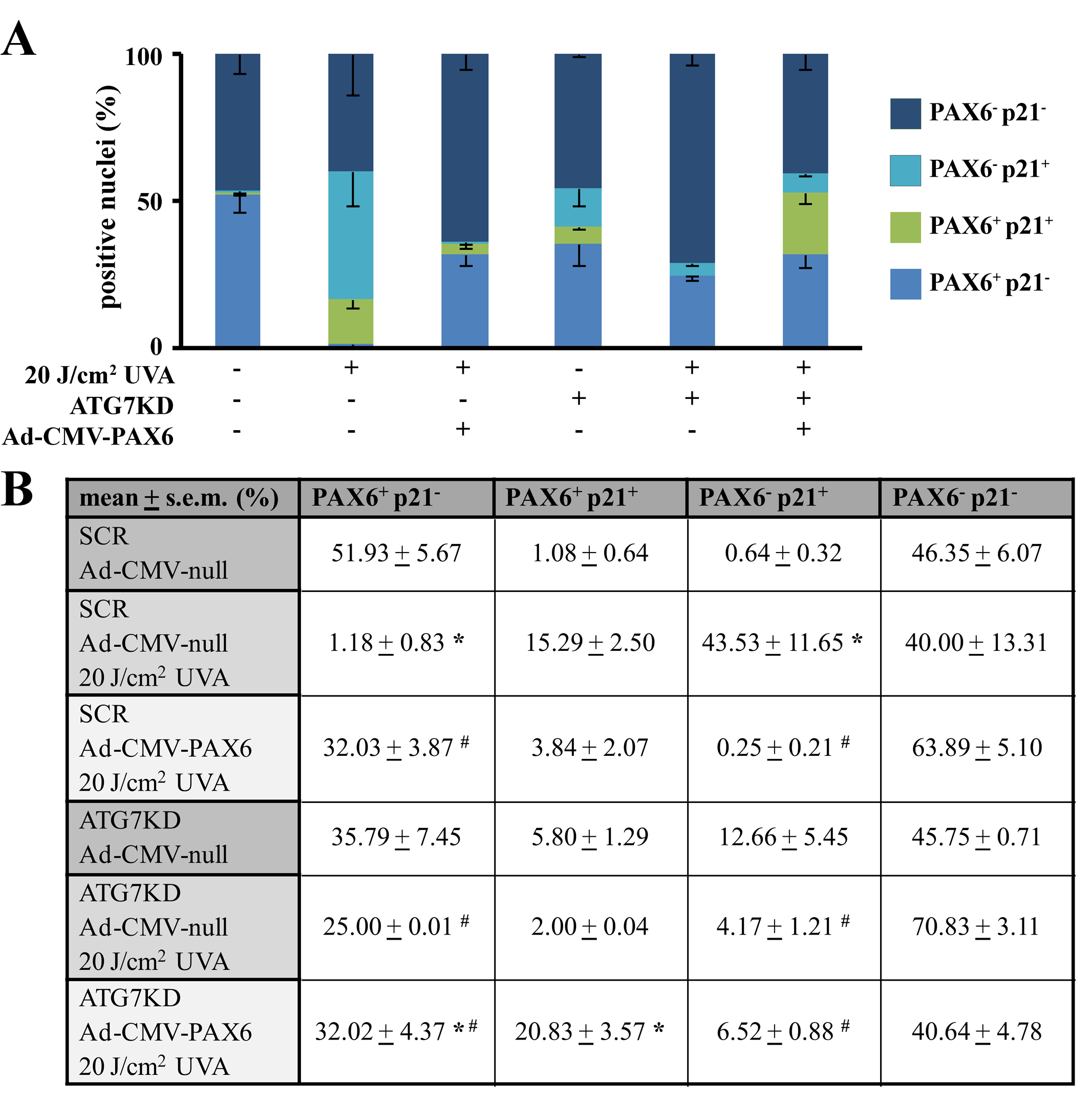

Supplement: S5 Fig — (A) Stacked bar diagram and (B) statistics of percentages of PAX6+p21-, PAX6+p21+, PAX6-p21+, and PAX6-p21- cells. *p < .05 compared to SCR, Ad-CMV-null controls, #p < .05 compared to SCR, Ad-CMV-null, UVA-irradiated counterparts. (TIF) [file pone.0180868.s005.tif]
